# BMP4 overexpression induces the upregulation of APP/Tau and memory deficits in Alzheimer’s disease

**DOI:** 10.1038/s41420-021-00435-x

**Published:** 2021-03-15

**Authors:** Xiaoqing Zhang, Juan Li, Li Ma, Hui Xu, Yun Cao, Wei Liang, Jia Ma, Z. Peter Wang, Yuyun Li

**Affiliations:** 1 Bengbu Medical College Key Laboratory of Cancer Research and Clinical Laboratory Diagnosis, Bengbu Medical College, 233030 Bengbu, Anhui China; 2 Department of Laboratory Medicine, School of Laboratory Medicine, Bengbu Medical College, 233030 Bengbu, Anhui China; 3 The Second People’s Hospital of Lianyungang, 222002 Lianyungang, Jiangsu China; 4 Department of Biochemistry and Molecular Biology, School of Laboratory Medicine, Bengbu Medical College, 233030 Bengbu, Anhui China

**Keywords:** Cellular neuroscience, Neurodegeneration

## Abstract

Alzheimer’s disease (AD) is a chronic progressive degenerative disease of the nervous system. Its pathogenesis is complex and is related to the abnormal expression of the amyloid β (Aβ), APP, and Tau proteins. Evidence has demonstrated that bone morphogenetic protein 4 (BMP4) is highly expressed in transgenic mouse models of AD and that endogenous levels of BMP4 mainly affect hippocampal function. To determine whether BMP4 participates in AD development, transgenic mice were constructed that overexpress BMP4 under the control of the neuron-specific enolase (NSE) promoter. We also performed MTT, FACS, transfection, TUNEL, and Western blotting assays to define the role of BMP4 in cells. We found that middle-aged BMP4 transgenic mice exhibited impaired memory via the Morris water maze experiment. Moreover, their hippocampal tissues exhibited high expression levels of AD-related proteins, including APP, Aβ, PSEN-1, Tau, P-Tau (Thr181), and P-Tau (Thr231). Furthermore, in multiple cell lines, the overexpression of BMP4 increased the expression of AD-related proteins, whereas the downregulation of BMP4 demonstrated opposing effects. Consistent with these results, BMP4 modulation affected cell apoptosis via the regulation of BAX and Bcl-2 expression in cells. Our findings indicate that BMP4 overexpression might be a potential factor to induce AD.

## Introduction

The misfolding and aggregation of certain proteins are fundamental features of neurodegenerative diseases, such as Alzheimer’s disease (AD). AD is an age-related, progressive, irreversible neurodegenerative disease that leads to the loss of selected neurons in the basal forebrain, almond nucleus, hippocampus, and cortex, as well as the progressive deceleration of cognition and memory^1–4^. The main manifestations of AD are progressive memory impairment, cognitive impairment, personality changes, language disorders, and other neurological symptoms^5^. The hippocampus is a critical brain region for learning and memory and is particularly vulnerable to damage in the early stages of AD. Emerging evidence suggests that neurogenesis in the adult hippocampus is an early critical event in the AD process^6^. Therefore, AD is characterized by the insidious development of hippocampal pathology^7^. The changes in neurogenesis observed during the initial stages and progression of AD have demonstrated that the modulation of the new production of neurons at neurogenic sites may exert profound effects on hippocampal function^1^. This pathological trait is linked to the deposition of amyloid β-protein (Aβ) peptides and the abnormal phosphorylation of Tau protein in the cerebral cortex and hippocampal formation^8,9^.

In AD, genetic evidence from mutations of the amyloid precursor protein (APP) and presenilins (PSENs) strongly suggests a role of Aβ in the pathogenesis of familial AD^10,11^. Aβ has three main forms, Aβ1-40, Aβ1-42 and Aβ 1-43, and Aβ 42/43 is a lamellar Aβ structure with strong hydrophobicity, easy deposition, and neurotoxicity. Under normal conditions, 90% of humans express primarily Aβ40 and only a small amount of Aβ42/43 in the brain, while in AD patients, the ratio of Aβ42/Aβ40 in the brain is unbalanced and increased, and Aβ42 deposits in the brain form the core of AD^12^. Tau, a microtubule-related protein, is found in neurons in the frontal temporal hippocampus and olfactory region of the brain. It has been shown to play a key role in the pathology of AD and is a potential target for the treatment of AD. Tau is a highly soluble and phosphorylated unstable substance. Approximately 20% (or 85 amino acid residues) of the amino acid sequence of the longest Tau subtype consists of potential (Ser, Thr, or Tyr) phosphorylation sites. The phosphorylation of the Tau protein in the normal brain remained at a low level, while the Tau phosphorylation level in AD patients is 3–4 times higher than that in normal people of the same age^13,14^.

BMPs perform their biological functions by interacting with membrane-binding receptors of the serine/threonine kinase family, including the BMP receptors type I (BMPRI) and type II (BMPRII)^15,16^. There are several types of BMPs in the hippocampus, such as BMP2, BMP4, BMP7, and BMP10^17^. BMPs or BMP-associated factors could regulate hippocampal plasticity and other important brain functions^18^. Bone morphogenetic protein 4 (BMP4), a member of the BMP family (BMPs), is part of the transforming growth factor-beta (TGF-β) protein superfamily. BMPs not only play physiological roles in embryonic hematopoietic and bone tissue but also regulate the production of neural stem cells in the equine region of the brain^19^. Precise control of the BMP4 signal in the extracellular space appears to play a key role in multiple events during development, including neural induction and changes in neurogenesis associated with functional changes in the hippocampus^20^. One study demonstrated that high levels of typical BMP signaling were detected in hippocampal niches^21^. In addition, ligands of the BMP family are involved in the regulation of resting adult hippocampal stem cells and in the control of progenitor cell maturation at multiple stages of the neurogenic lineage^22^. Changes in neurogenesis in the subgranular area of the hippocampal dentate gyrus (DG) have been demonstrated in various animal models of AD and in patients with AD. These changes usually cause working memory and cognitive impairment in AD. Another study showed that BMP4 inhibits nerves in the hippocampus in certain behavioral disorders primarily associated with AD^23^.

Although these studies indicated that BMP4 might be involved in AD development and progression, there is no direct evidence to reveal the function of BMP4 in AD development. To determine whether BMP4 is a driver of AD, transgenic mice were constructed overexpressing BMP4 under the control of the neuron-specific enolase (NSE) promoter. We also determined whether BMP4 modulation in multiple cell lines regulated the expression of AD biomarkers, such as APP, PSEN-1, and Tau, and led to the regulation of cell apoptosis. Our study will provide evidence for the mechanisms of BMP4 participation in AD development.

## Results

### Construction and screening of NSE-BMP4 transgenic mice

**Fig. 1:**
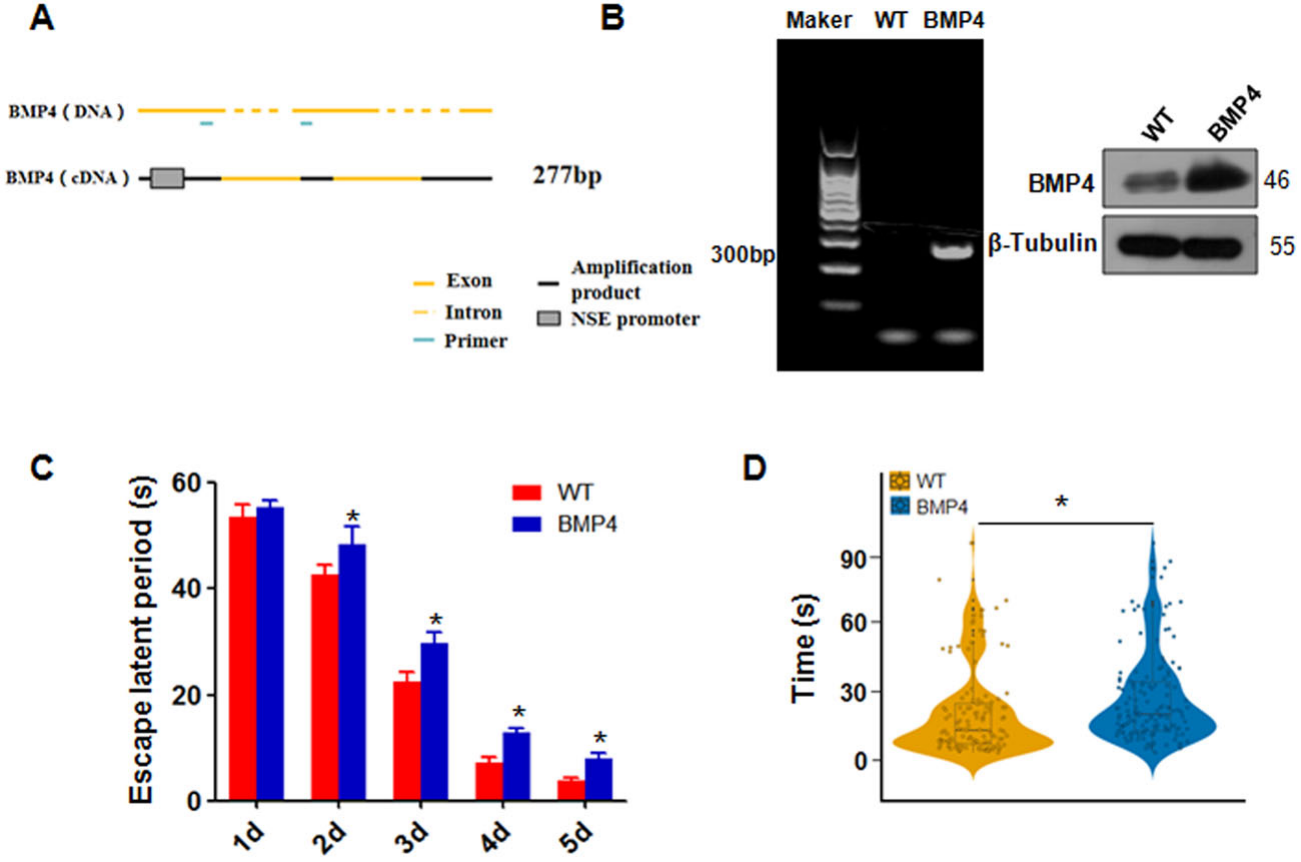
The escape latency of NSE-BMP4 transgenic mice is longer. **A** The primers for BMP4 are illustrated to show the crossed introns. **B** Left panel: RT-PCR analysis was used to measure NSE-BMP4 transgene expression in the adult transgenic hippocampus. Right panel: Western blotting was utilized to measure the BMP4 expression in the hippocampus of wild-type and adult transgenic mice. **C** The Morris water maze experiment was conducted in elderly mice. Each group has ten mice. *n* = 10. *P < 0.05 vs. WT. WT: wild-type mice. BMP4: BMP4 transgenic mice. **D** The total distribution of the five-day escape incubation periods in different groups. *P < 0.05 vs. WT.

We used C57BL/6 mice to construct a BMP4 (NSE-BMP4) transgenic model regulated by the NSE promoter. Under the regulation of the NSE promoter, the expression level of BMP4 was upregulated. The mouse BMP4 gene sequence includes exons and introns. The primers for BMP4 were designed to cross introns, resulting in a specific amplification band of approximately 277 bp (Fig. 1A). RT-PCR analysis data revealed the expression of the transgene in the NSE-BMP4 forebrain and hippocampus of mice at 4 weeks of age (Fig. 1B and Supplementary Fig. 1A). Moreover, western blotting data confirmed the high expression of BMP4 protein in the hippocampus of the transgenic mice (Fig. 1B). Furthermore, BMP4 transgenic mice had fewer BrdU^+^/NeuN^+^ neurons in the dentate gyrus of the hippocampus than wild-type controls (Supplementary Fig. 1B). Thus, we have constructed transgenic mice overexpressing BMP4 under the control of the NSE promoter, and we used these mice to determine the function of BMP4 in brain development and AD progression.

### The escape latency of NSE-BMP4 transgenic mice is longer

To determine whether the NSE-BMP4 transgenic mice suffer from AD, a morris water maze experiment was conducted in elderly mice (30 weeks of age). The results showed that there was no significant difference in the escape latency of the water maze between NSE-BMP4 transgenic mice and wild-type mice on the first day, but there was a marked difference (*p* < 0.01) on the 2nd to 5th day(Fig. 1C). The total distribution of the five-day escape incubation period in the different groups (Fig. 1D) showed that there was a difference between the transgenic group and the wild-type group. This finding indicates that BMP4 overexpression might involve the regulation of cognitive function in mice.

### BMP4 transgenic mice exhibit higher expression of AD-related proteins

**Fig. 2:**
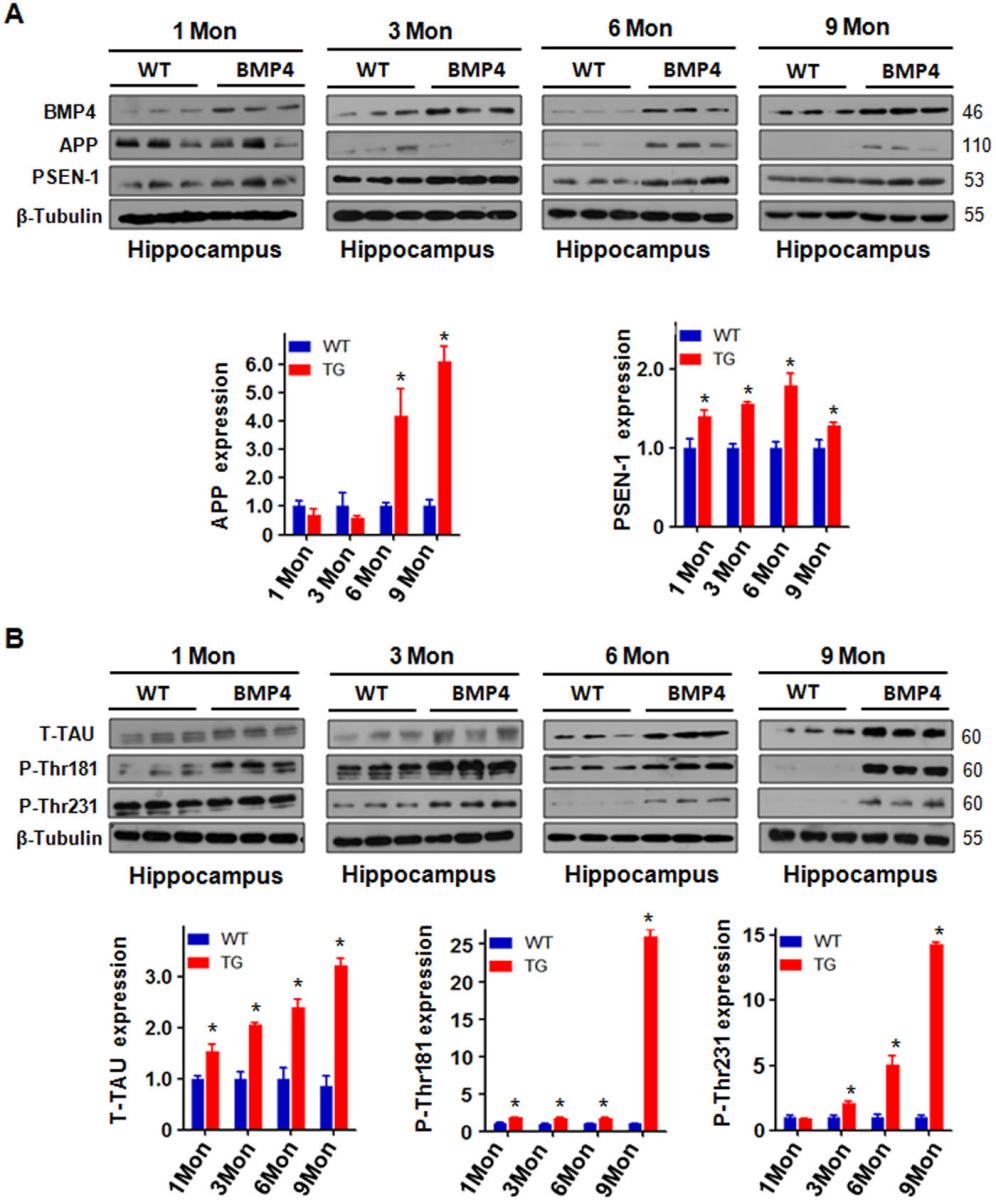
BMP4 transgenic mice exhibit higher expression of AD-related proteins. **A** Top panel: Western blotting analysis was used to measure the expression of BMP4, APP, and PSEN-1 in the hippocampus of BMP4 transgenic mice and WT mice. Bottom panel: Quantitative results are illustrated for the top panel. *P < 0.05 vs. WT. WT: wild-type mice; BMP4: BMP4 transgenic mice. **B** Top panel: Western blotting analysis was performed to measure the expression of T-Tau, P-Thr181 Tau, and P-Thr231 Tau in the hippocampus of BMP4 transgenic mice and WT mice. Bottom panel: Quantitative results are illustrated for the top panel. *P < 0.05 compared with WT. β-Tubulin as an internal loading control was used for normalization. The relative expression of proteins in BMP transgenic mice was compared with WT mice at each point.

Next, we extracted hippocampal tissue proteins from NSE-BMP4 transgenic mice and wild-type mice at 1, 3, 6, and 9 months of age and compared the differences in APP family-related proteins by Western blotting. We found that the expression of APP and PSEN1 was promoted by BMP4 in transgenic mice at 6 months of age (Fig. 2A). The results showed that there was no difference in the expression of APP family-related proteins between the one-month-old NSE-BMP4 transgenic mice and the wild-type mice (Fig. 2A). There was a decrease in APP protein level at 3 months compared with that at 1 month, which means that APP is being sheared into Aβ and is beginning to be deposited. In addition, BMP4 overexpression did not change the protein level of APP at 1 month and 3 months of age of transgenic mice (Fig. 2A), indicating that BMP4 overexpression-induced Alzheimer’s disease needs a progressive process. Moreover, the Western blotting data showed that the expression of T-Tau, p-Thr181 Tau, and p-Thr231 Tau in mice with BMP4 overexpression was significantly higher than that in wild-type mice (Fig. 2B). Furthermore, the older the mice were, the more obvious the increase in these proteins was (Fig. 2B).

### Overexpression of BMP4 promotes the expression of AD-related proteins in cell lines

**Fig. 3:**
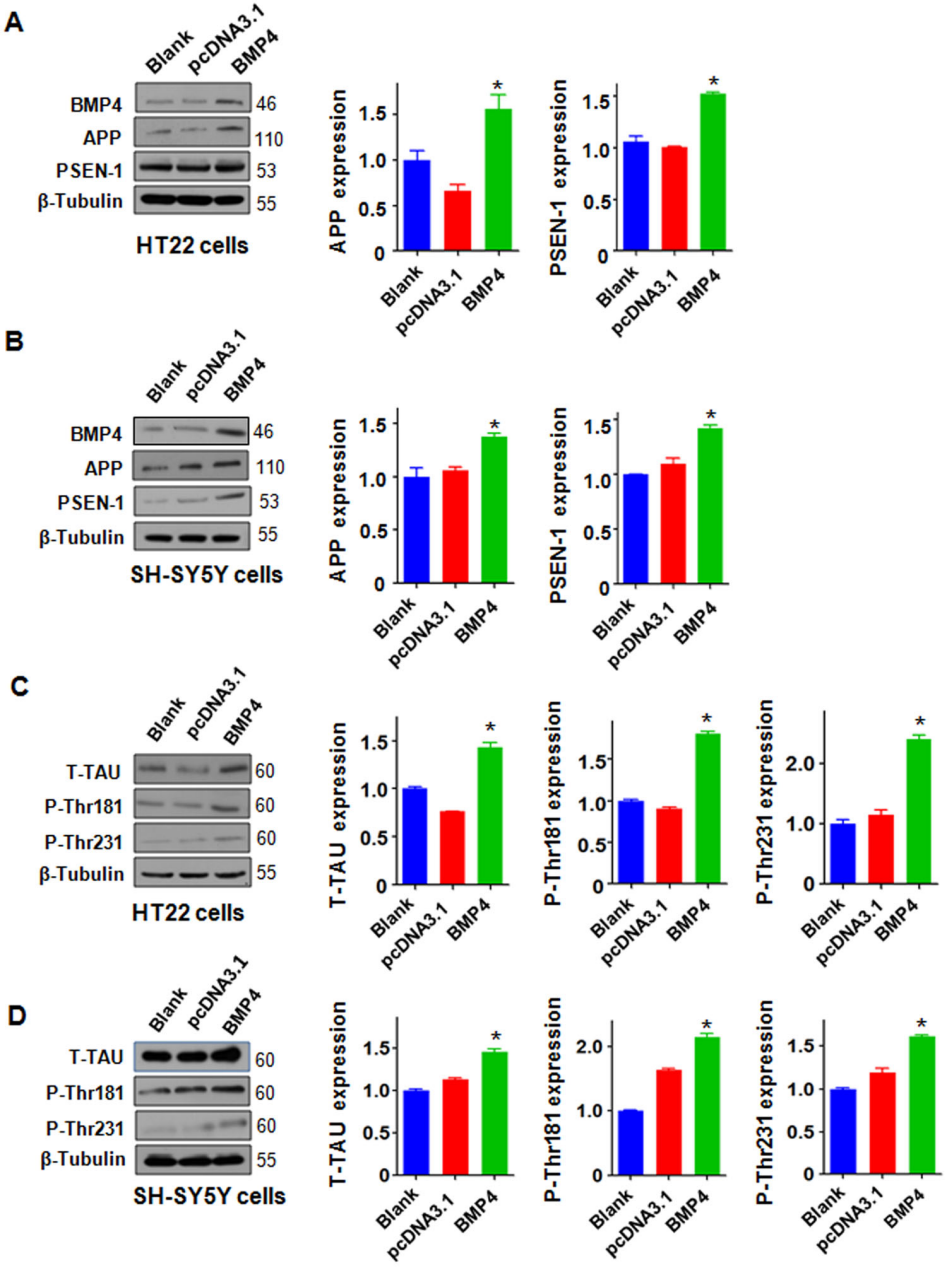
Overexpression of BMP4 promotes the expression of AD-related proteins in cell lines. **A–B** Left panel: Western blotting analysis was performed to measure the expression of APP and PSEN-1 in HT22 cells (**A**) and SH-SY5Y cells (**B**) transfected with the BMP4 overexpressing plasmid. Right panel: Quantitative results are illustrated for the left panel. *P < 0.05 vs. pcDNA3.1. **C–D** Left panel: Western blotting analysis was performed to measure the expression of T-Tau, P-Thr181 Tau, and P-Thr231 Tau in the HT22 cells (**C**) and SH-SY5Y cells (**D**) transfected with the BMP4 overexpressing plasmid. Right panel: Quantitative results are illustrated for the left panel. *P < 0.05 vs. pcDNA3.1.

To further define whether BMP4 modulates the expression of AD-related proteins, we performed in vitro experiments using HT22, N2A, and SH-SY5Y cells. The cells were transfected with a BMP4 overexpression plasmid, and the cell proteins were extracted after 48 h. Our Western blotting data showed that the overexpression of BMP4 promoted the expression of APP and PSEN1 in the HT22 (Fig. 3A), SH-SY5Y (Fig. 3B), and N2A cells (Supplemental Fig. 2A). Moreover, the overexpression of BMP4 increased the expression of T-Tau, p-Thr181 Tau, and p-Thr231 Tau in the HT22 (Fig. 3C), SH-SY5Y (Fig. 3D), and N2A cells (Supplemental Fig. 2B). These data show that BMP4 overexpression could govern the expression of AD-related proteins.

### Downregulation of BMP4 reduces the expression of AD-related proteins

**Fig. 4:**
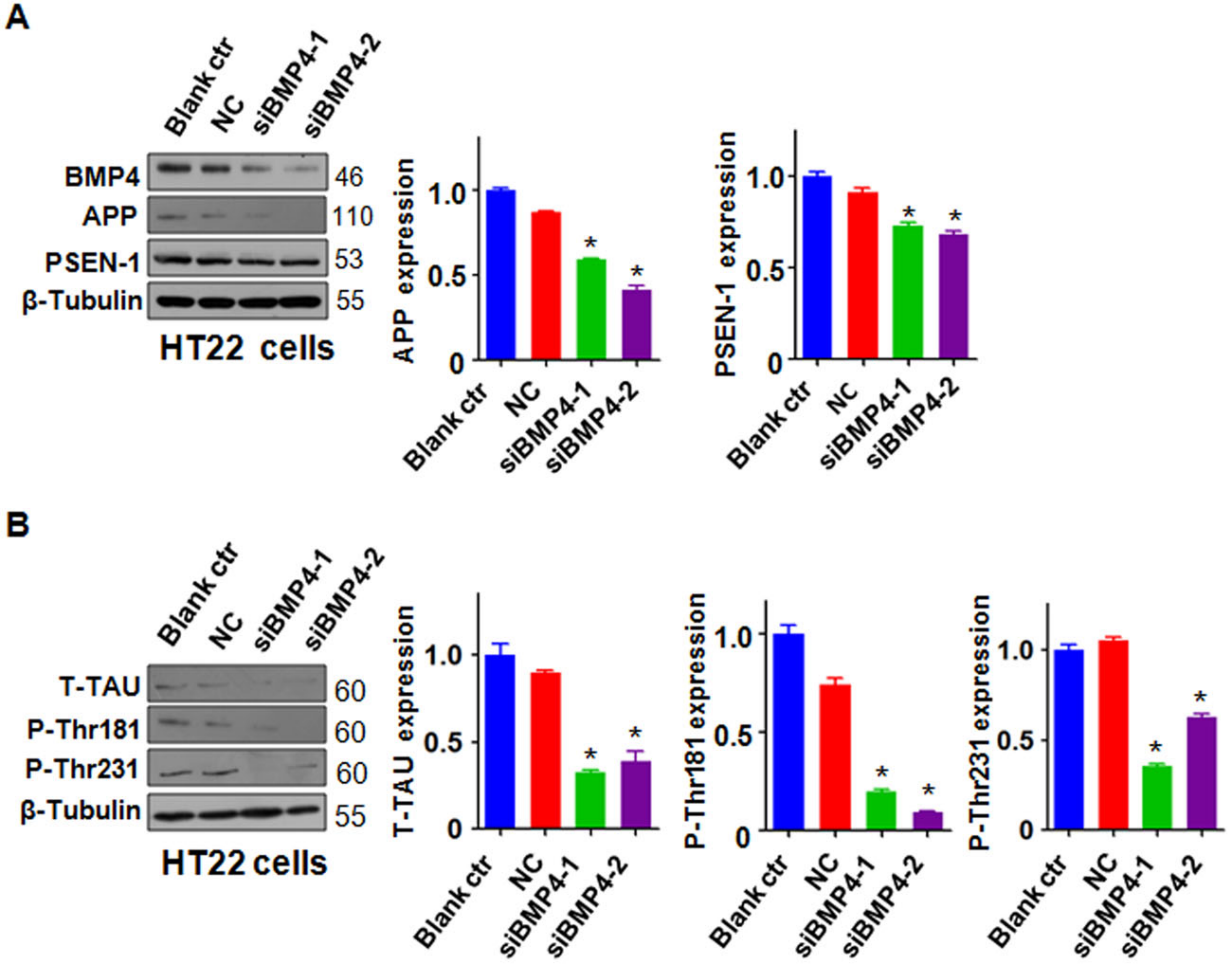
Downregulation of BMP4 reduces the expression of AD-related proteins. **A** Left panel: Western blotting analysis was performed to measure the expression of APP and PSEN-1 in HT22 cells transfected with BMP4 siRNAs. Right panel: Quantitative results are illustrated for the left panel. *P < 0.05 vs. NC. NC: nonspecific control. **B** Left panel: Western blotting analysis was performed to measure the expression of T-Tau, P-Thr181 Tau, and P-Thr231 Tau in HT22 cells transfected with BMP4 siRNAs. Right panel: Quantitative results are illustrated for the left panel. *P < 0.05 vs. NC.

To further investigate whether BMP4 governs the expression of AD-related proteins, HT22 and N2A cells were transfected with BMP4-specific small interfering RNA (siRNA) to downregulate BMP4. We found that the BMP4 siRNA decreased the expression of BMP4 in the HT22 (Fig. 4A) and N2A cells (Supplemental Fig. 3A). Notably, the downregulation of BMP4 weakened the expression of APP and PSEN1 proteins in the HT22 and N2A cells (Fig. 4A and Supplemental Fig. 3A). Furthermore, the inhibition of BMP4 decreased the expression of T-Tau, p-Thr181 Tau, and p-Thr231 Tau in the HT22 cells (Fig. 4B) and N2A cells (Supplemental Fig. 3B). Taken together, these data indicate that BMP4 might control the expression of AD-related proteins.

### BMP4 overexpression induces BAX expression and reduces Bcl-2 levels

**Fig. 5:**
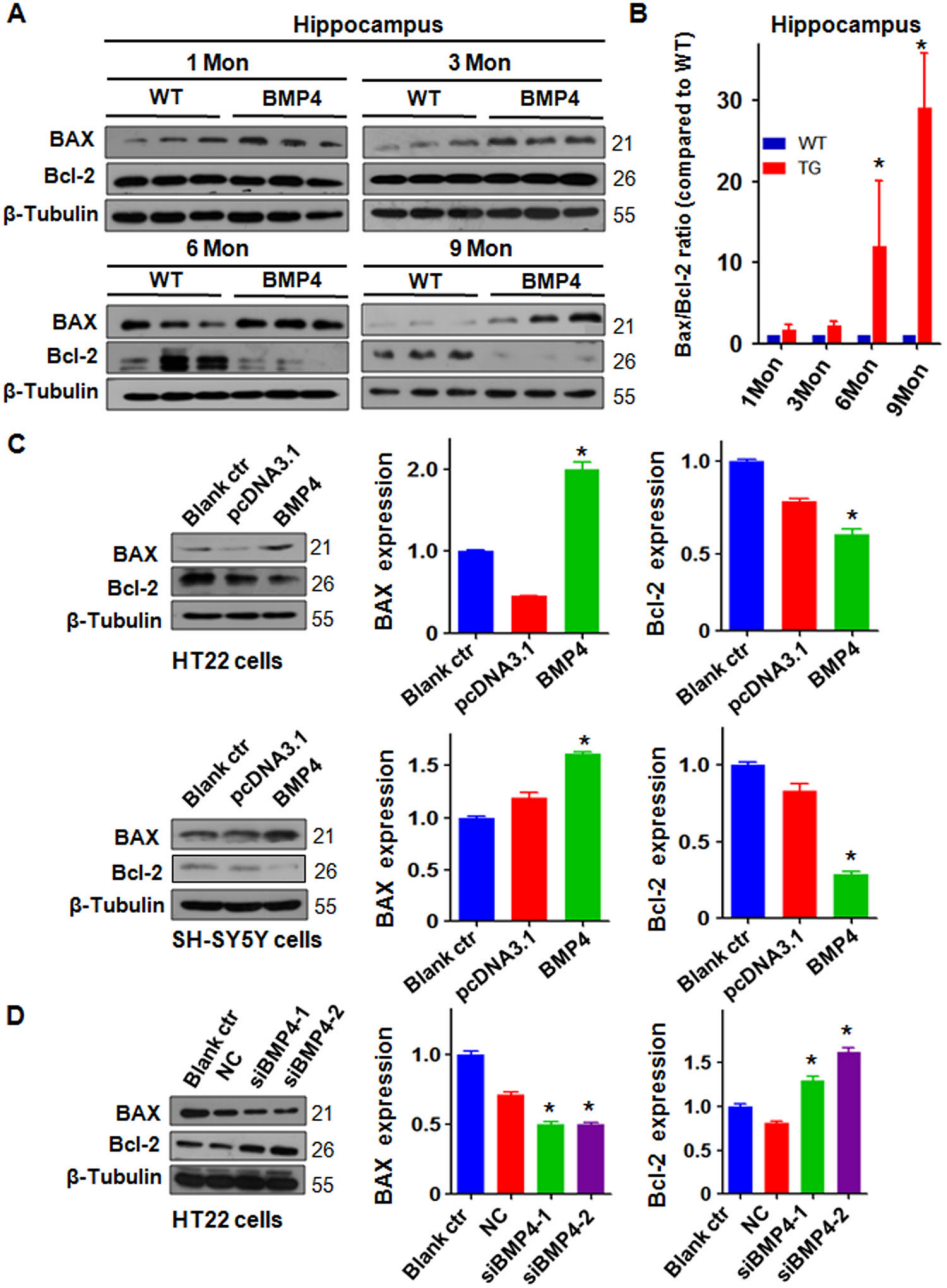
BMP4 overexpression induces BAX expression and reduces Bcl-2 levels. **A** Western blotting analysis was used to measure the expression of BAX and Bcl-2 in the hippocampus of BMP4 transgenic mice and WT mice. **B** Quantitative results of BAX/Bcl-2 are illustrated for panel **A**. *P < 0.05 vs. WT. WT: wild-type mice. TG: BMP4 transgenic mice. β-Tubulin as an internal loading control was used for normalization. The relative expression of BAX/Bcl-2 in BMP transgenic mice was compared with WT mice at each point. **C** Left panel: Western blotting analysis was performed to measure the expression of BAX and Bcl-2 in HT22 and SH-SY5Y cells transfected with the BMP4 overexpression plasmid. Right panel: Quantitative results are illustrated for the left panel. *P < 0.05 vs. pcDNA3.1. **D** Left panel: Western blotting analysis was performed to measure the expression of BAX and Bcl-2 in HT22 cells transfected with BMP4 siRNAs. Right panel: Quantitative results are illustrated for the left panel. *P < 0.05 vs. NC.

To explore whether the overexpression of BMP4 induces apoptosis and is involved in the development of AD, we extracted hippocampal proteins from aged NSE-BMP4 mice and wild-type mice and measured the expression of Bcl-2 and BAX by Western blotting. We found that the BMP4 transgenic mice exhibited increased BAX and downregulated Bcl-2 at 6 and 9 months (Fig. 5A, B). The in vitro experiments demonstrated that the overexpression of BMP4 increased the expression of BAX and decreased Bcl-2 expression in the HT22 and SH-SY5Y cells (Fig. 5C) and N2A cells (Supplemental Fig. 4A). In line with these results, the downregulation of BMP4 reduced BAX expression and increased Bcl-2 expression levels in the HT22 cells (Fig. 5D) and N2A cells (Supplemental Fig. 4B). Therefore, BMP4 overexpression induced apoptosis in part via regulating BAX and Bcl-2 expression.

### BMP4 overexpression induces apoptosis in cells

**Fig. 6:**
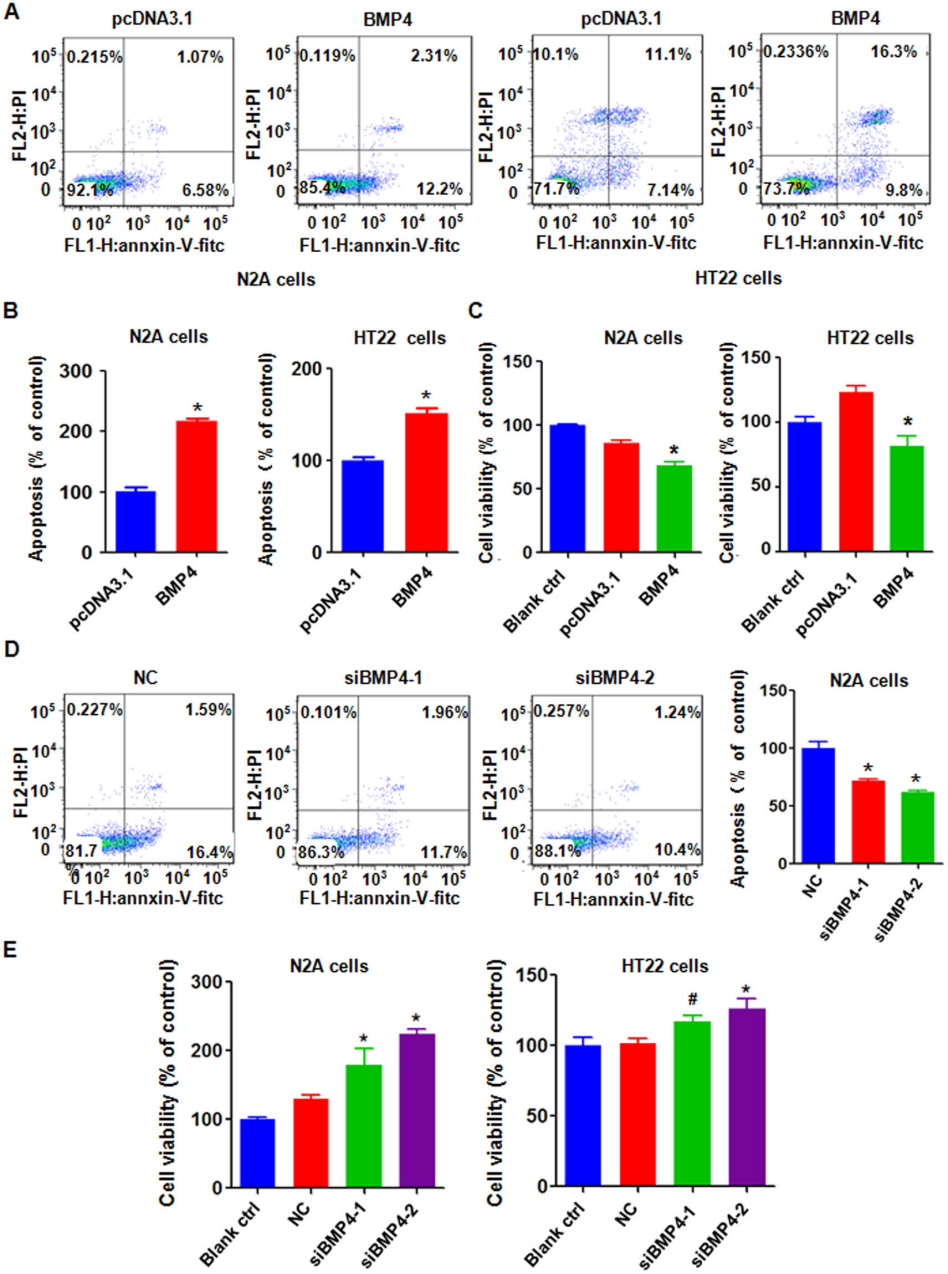
BMP4 overexpression induces apoptosis in cells. **A** Cell apoptosis was tested via flow cytometry in N2A and HT22 cells with overexpressing BMP4. **B**. Quantitative results are illustrated for panel **A**. *P < 0.05, compared with pcDNA3.1. **C** The MTT assay was performed to measure cell viability in N2A and HT22 cells overexpressing BMP4. *P < 0.05, compared with pcDNA3.1. **D** Left panel: Cell apoptosis was tested via flow cytometry in N2A cells transfected with BMP4 siRNA. Right panel: Quantitative results are illustrated for the left panel. *P < 0.05, compared with NC. NC: nonspecific control siRNA. siBMP4: BMP4 siRNA. **E** The MTT assay was used to test cell viability in N2A and HT22 cells transfected with BMP4 siRNA.

Next, we dissected whether BMP4 overexpression is involved in apoptosis in cell lines. We observed that the overexpression of BMP4 induced apoptotic cell death in the N2A and HT22 cells (Fig. 6A, B). We also found that BMP4 upregulation inhibited cell viability in the N2A and HT22 cells (Fig. 6C). Consistent with these data, the downregulation of BMP4 inhibited cell apoptosis in the N2A cells (Fig. 6D) and promoted cell viability in the N2A and HT22 cells (Fig. 6E).

### Apoptosis is increased in the DG, CA1, and CA3 of BMP4 transgenic mice

**Fig. 7:**
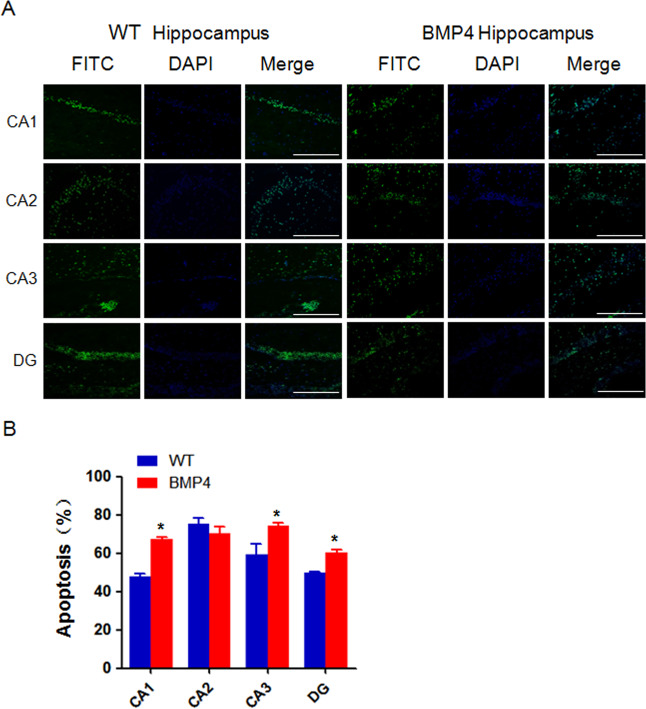
Apoptosis is increased in the DG, CA1, and CA3 of BMP4 transgenic mice. **A** Apoptosis was measured in the DG region and CA1-3 zones of the hippocampus in 9-month-old wild-type and NSE-BMP4 transgenic mice by TUNEL fluorescence assay. Each group has five mice. *n* = 6. Bar: 50 μM. **B** Quantitative results are illustrated for panel **A**. *P < 0.05, compared with WT.

Finally, we measured apoptosis in the DG region and the CA1-3 zones of the hippocampus in 9-month-old wild-type and NSE-BMP4 transgenic mice by TUNEL fluorescence assay. We found that the CA1, CA3, and DG zones in the transgenic mice exhibited more apoptosis than those of the wild-type mice (Fig. 7A–B). Cell rearrangement and evacuation of CA1, CA2, CA3 cells were observed in the transgenic mice and the boundaries were not clear. These results indicate that the NSE-BMP4 mice experience neuronal cell loss (Fig. 7A).

## Discussion

In this study, we explored the role of BMP4 in AD development and progression. We found that the middle-aged BMP4 transgenic mice exhibited memory impairment via the Morris water maze experiment. Moreover, the hippocampal tissues exhibited high expression of AD-related proteins including APP, PSEN-1, Tau, p-Tau (Thr181), and p-Tau (Thr231). Furthermore, in multiple cell lines, the overexpression of BMP4 increased the expression of AD-related proteins, whereas the downregulation of BMP4 demonstrated opposing effects. Consistently, BMP4 modulation participated in cell apoptosis via the regulation of BAX and Bcl-2 expression in cells. Our findings indicate that BMP4 overexpression might be a potential factor that participates in AD.

In recent years, with the gradual improvement of living standards and the aging of the population, the number of AD patients has increased, leading to AD becoming a global health priority^24^. The main manifestations of AD are progressive memory impairment, cognitive impairment, personality changes, language disorders, and other neurological symptoms^5^. The hippocampus is a critical brain region for learning and memory and is particularly vulnerable to damage in the early stages of AD. Emerging evidence suggests that neurogenesis in the adult hippocampus is an early critical event in the AD process^6^. BMPs perform their biological functions by interacting with membrane-binding receptors of the serine/threonine kinase family, including the BMP receptors type I (BMPRI) and type II (BMPRII). There are several types of BMPs in the hippocampus, such as BMP2, BMP4, BMP7, and BMP10. This suggests that BMPs or BMP-associated factors could regulate hippocampal plasticity and other important brain functions. Upregulation of BMP6 has been observed in the brains of AD patients and APP transgenic mice and is associated with impaired neurogenesis^25,26^. BMP-9 was reported to affect some aspects of AD and two small peptides derived from BMP-9 (pBMP-9 and SpBMP-9) induced the cholinergic differentiation via activation of PI3K/Akt and inactivation of GSK3β in SH-SY5Y neuroblastoma cells^27^. Moreover, this SpBMP-9 peptide enhanced the functions of bFGF and NGF on SH-SY5Y cell differentiation^28^. Physical activity protects the AD-induced pathological alterations in the kidneys in part via upregulation of pituitary adenylate cyclase-activating polypeptide (PACAP) and BMP4 signaling pathway^29^. One study proved that BMP4 is strongly expressed in the subgranular layer of the adult hippocampal dentate gyrus (DG)^23^. The RNA level of BMP4 was shown to be increased in the hippocampus of APPswe/PS1 transgenic mice, linking BMP4 with AD development, but little is known about the mechanism through which BMP4 is involved in the occurrence of AD^23^. In this study, we used an NSE-BMP4 transgenic mouse model to determine whether BMP4 overexpression could trigger the development of AD. In fact, BMP4 transgenic mice exhibited memory impairments in the Morris water maze experiment, indicating that BMP4 overexpression might be involved in AD pathogenesis.

AD is often caused by mutations in the APP gene, and APP dysfunction occurs prior to Tau dysfunction^30^. Mutations in the Tau gene lead to dominant dementia and Parkinson’s disease^8^, and Aβ may play its role in patients with a cognitive impairment through Tau^31^. Moreover, the deposition of Tau in the cortex and hippocampus is conducive to the occurrence and development of AD, although it is not an essential factor for AD development^8^. It has been recognized that Aβ1-42, T-Tau, and P-Thr181 Tau are auxiliary indicators of AD^32–34^. T-tau and P-Thr181 Tau have been identified as core neurochemical AD biomarkers^35–37^. P-Thr231 Tau appears to be more closely associated with AD^38–41^. Therefore, in this study, we determined the role of T-Tau, P-Thr181 Tau, and P-Thr231 in AD development. BMP4 has a function during neurogenesis in the adult hippocampus in AD^23^. It has been reported that APPswe/PS1δE9 mice of AD had overexpression of BMP4 in hippocampal DG zone, indicating that increased BMP4 might contribute to suppression of hippocampal cell proliferation^42^. Moreover, BMP4 and Noggin co-modulated adult hippocampal neurogenesis in this AD mouse model^43^. Furthermore, APP transgenic mice had an upregulation of BMP4 and p-Smad1/5/8^44^. In line with these findings, BMP4 transgenic mice exhibited higher expression levels of AD-related proteins including APP, T-Tau, P-Thr181 Tau, and P-Thr231 Tau. Further investigations are required to determine whether other phospohoepitopes of Tau, such as P-Thr217, are involved in BMP4-mediated AD. Interestingly, the expression of APP protein was decreased in both WT and BMP4 transgenic mice at 3 months compared with its expression in WT and BMP4-overexpressing mice at 1 month. Moreover, the expression of APP was upregulated in BMP4 transgenic mice at 6 months and 9 months compared with its expression at 3 months, while APP expression has no remarkably changed in WT mice at 3, 6, and 9 months. Similarly, the expression of P-Thr231 Tau exhibited a dynamic change during 1 month to 9 months in BMP4 transgenic mice. These dynamic changes of APP and P231 Tau in BMP4 transgenic mice need to be in-depth investigated. Consistently, the overexpression of BMP4 in cell lines promoted the expression of AD-associated proteins. Since BMP4 knockout leads to lethality in mice^45,46^, we knocked down BMP4 in multiple neuroma cell lines. We found that the downregulation of BMP4 in the cell lines reduced the expression of AD-related proteins. Our data implicated that BMP4 might be related to AD development.

Bcl-2 family proteins, including pro-apoptotic and anti-apoptotic members, are involved in cell apoptosis in AD^47^. In our study, the BMP4 transgenic mice exhibited increased expression of BAX and reduced Bcl-2 levels. The overexpression of BMP4 increased BAX levels and inhibited Bcl-2 expression in cell lines, indicating that BMP4 overexpression induces AD in part via regulation of BAX and Bcl-2. This study used a conditional BMP4 transgenic mouse model and found that BMP4 overexpression led to memory impairment in mice. However, there are several limitations of this study. In addition to the Morris water maze experiment, could any other experiments by assessing cognitive functions to detect behavioral changes validate AD development in mice overexpressing BMP4? Do AD patients in the clinic express higher levels of BMP4? What are the detailed molecular mechanisms of BMP4-involved AD development? These questions need to be answered in the future to fully elucidate the role of BMP4 in AD initiation and progression. In summary, upregulation of BMP4 might increase the expression of AD-related proteins and subsequently elevate the BAX/Bcl-2 ratio, leading to cell death and AD development.

## Methods

### Animals

C57BL/6 mice were bred in the SPF grade animal barrier system in Bengbu Medical College Experimental Animal Center. Wild-type (WT) mice were purchased from the experimental animal center of Yangzhou University (Yangzhou, Jiangsu, China). The NSE-BMP4 transgenic mice were derived from Northwestern University (Chicago, IL, USA). All animal experiments were conducted in accordance with the rules and regulations of the Animal Ethics Committee of Bengbu Medical College.

### Reagents

The DNA kit was purchased from Foregene Company (Chengdu, Sichuan, China). The MTT (3-(4,5-dimethyl-2-thiazolyl)-2,5-diphenyl-2-H-tetrazolium bromide) was bought from Sigma-Aldrich (St. Louis, MO, USA). The PCR primers were synthesized by and purchased from Genscript Company (Nanjing, Jiangsu, China). The agarose gel was purchased from Biowest Company. The TRIzol ® Reagent and DNA ladder were acquired from TIANGEN (Tianjin, China). The SYBR Premix Dimer Eraser and Reverse Transcription kit were purchased from Takara (Japan). The 40 μm cell filters were obtained from Biologix Company (Shanghai, China). The Lipofectamine 3000 reagent was obtained from Invitrogen (Waltham, MA, USA). The BSA was purchased from Biofroxx Biotechnology (Germany). The BCA kit and SDS-PAGE kit were purchased from Beyotime Biotechnology (Shanghai, China). The anti-BMP4 (ab39973) and anti-P-tau231 (ab151559) antibodies were obtained from Abcam (Cambridge, MA, USA). The anti-APP (#29765), anti-Tau (#4019), and anti-P-Tau181 (#12885) antibodies were obtained from Cell Signaling Technology (Danvers, MA, USA). The anti-BAX (50599-2-Ig), anti-Bcl-2 (26593-1-AP), and anti-PSEN1 (16163-1-AP) antibodies were obtained from ProteinTech Company (Wuhan, Hubei, China).

### Cell culture

The hippocampal neurons (HT22) cell line, neuroblastoma (N2A) cell line, and human neuroblastoma (SH-SY5Y) cells were purchased from ATCC Company (Manassas, VA, USA) with short-tandem repeat profiling for authentication. The cells were grown in DMEM (Dulbecco’s modified Eagle medium, Gibco Invitrogen) supplemented with 10% fetal bovine serum (FBS) and 1% penicillin and streptomycin. The cells were maintained in a 5% CO_2_ culture incubator at 37 °C.

### Breeding and screening of BMP4 transgenic mice

Adult NSE-BMP4 and WT mice (8 weeks) were placed in cages with a male-female ratio of 1:1 or 1:2, and vaginal plug were detected 12 h later. The pregnant female mice were identified, and the marked time was 0.5 days of gestation (E0.5). After 21 days, the mother mice finished pregnancy and gave birth to pups. When the pups reached 3 weeks of age, tail samples approximately 3 mm in the length of the mouse were collected to extract DNA for PCR amplification and DNA gel electrophoresis to identify the BMP4-positive transgenic mice.

### Morris water maze assay

Ten transgenic mice with BMP4 overexpression and ten WT mice were randomly selected for the Morris water maze experiment at 30 weeks of age. The Morris water maze pool was divided into four quadrant areas: I, II, III, and IV. The platform was placed in the middle of the third quadrant 40 cm from the pool wall, and the water surface was 1 cm above the top of the platform to ensure the mice were unable to see the platform from the water surface. The temperature was maintained at 22–24 °C. The test time was 60 s, and staying on the platform for more than 2 s was considered the successful location of the platform. After 5 days of experimental training, the time required by the mice after entering the water to successfully locate the platform was recorded as the escape incubation period, and the escape incubation period was denoted as 60 s when the platform search failed. The averages of the results of 4 training sessions every day were taken as the total results of the training that day.

### Immunofluorescence

The immunofluorescence assay was performed as described previously^48^. The brain sections were cut in a cryostat and were incubated with 8% serum for 2 h at room temperature, then incubated with primary antibodies against BrdU (1:200; Abcam) and NeuN (1:200, CST) overnight at the cold room. After 1 h incubation with anti-mouse IgG/Alexa Fluor 488 or anti-rabbit IgG/Alexa Fluor 594, DAPI was used to counterstain for 10 min. The images were obtained under a fluorescence microscope.

### Transfection

BMP4 siRNA was purchased from Genepharma (Shanghai, China). BMP4 plasmids were purchased from Youbio (Changsha, Hunan, China). N2A, HT22, and SH-SY5Y cells were grown in 6-well plates and then transfected with the BMP4 overexpressing plasmid or empty vector using Lipofectamine 3000. The cells were also transfected with BMP4 siRNA and negative control using Lipofectamine 2000 according to the manufacturer’s instructions.

### Reverse-transcribed polymerase chain reaction

The level of BMP4 in the forebrain and hippocampus tissues was evaluated by reverse-transcribed polymerase chain reaction (RT-PCR). The total RNA was first extracted using TRIzol reagent. The level of BMP4 was measured by RT-PCR. The primers of BMP4: 5’-CAC TGT GAG GAG TTT CCA ATC-3’ and 5’-GTG ATG GAC TAG TCT GGT GTC-3’. Glyceraldehyde 3-phosphate dehydrogenase (GAPDH) was used as the internal reference.

### Western blot analysis

The hippocampal tissues of transgenic and wild-type mice aged 4, 12, 20, and 40 weeks were lysed with RIPA buffer with a 1% phosphatase inhibitor to extract the proteins. The N2A, HT22, and SH-SY5Y cells were harvested after transfection, and the total proteins from the cells were extracted. The protein samples were boiled and electrophoresed on 10% polyacrylamide gels and transferred onto polyvinylidene fluoride (PVDF) membranes. The membranes were blocked with 5% BSA in TBST (Tris-buffered saline with Tween) at room temperature for 2 h and then incubated with primary antibodies overnight at 4 °C. After washing with TBST, the membranes were incubated with HRP-conjugated goat anti-rabbit or mouse anti-rabbit secondary antibodies for 1 h at room temperature. Finally, the chemiluminescence reagent was added to the membranes and the membranes were exposed in the darkroom. The densitometric quantification of the protein blots was performed using ImageJ software. β-Tubulin as an internal loading control was used for normalization. The relative expression of proteins in BMP4 transgenic mice was compared with WT mice at each point.

### Cell viability assay

N2A, HT22, and SH-SY5Y cells were cultured in 96-well plates overnight, and then cells were transfected with the BMP4 overexpressing plasmid or the BMP4 siRNA for 72 h. Cell proliferation was assessed by the MTT assay^49^.

### Apoptosis assay

N2A, HT22, and SH-SY5Y cells (5 × 10^5^ cells/well) were seeded in a 6-well plate overnight and then transfected with BMP4 overexpressing plasmid or the BMP4 siRNA. After 24 h, cell apoptosis was assessed by FITC-Annexin/propidium iodide (PI) staining and flow cytometry. The cells were trypsinized and washed with PBS, and then resuspended in 500 μL binding buffer containing 1 μL FITC-conjugated anti-Annexin V antibody and 5 μL PI. Fluorescence-activated cell sorting (FACS) was performed to analyze the apoptotic rate. The FACS data were analyzed with the FlowJo software.

### Tissue processing

After the mice were killed, the scalp was cut open, and the skull was exposed. Then, the skull was removed with straight tweezers to expose the cerebral cortex, and the cerebral cortex was carefully removed with straight tweezers to expose the hippocampal tissue. The hippocampal tissue was separated from the cerebral cortex and the surrounding brain tissue and removed. Then, the samples were snipped with scissors and placed in a homogenizer for cell lysis, and the corresponding amount of RIPA was added to obtain the hippocampal tissue proteins.

### TUNEL assay for measuring cellular apoptosis in the mouse brain

The TdT-mediated dUTP-biotin nick end-labeling (TUNEL) assays were performed by using the TUNEL cell apoptosis detection kit via a green FITC-labeled fluorescence detection approach (catalog: KGA7071, KeyGEN BioTECH Company). Mouse cardiac perfusion was performed to make paraffin slides containing mouse hippocampal tissue. After dewaxing the paraffin sections of mouse hippocampal tissue, 100 μL (20 μg/mL) of the proteinase K working solution was added and washed with PBS 3 times at room temperature for 30 min. Then, the slide was incubated in 100 μL DNase I at 37 °C for 30 min and washed with PBS. After the slide was dried, 50 μL TUNEL reaction mixture (50 μL TdT + 450 μL fluorescein-labeled d UTP solution) was added to the specimens, which were incubated at 37 °C for 60 min and then washed with PBS. DAPI was added dropwise to protect the specimens for 10 min, and the specimens were stained and washed with PBS. The excess DAPI was washed off, the liquid on the slide was blotted with absorbent paper, and the image was observed under a fluorescence microscope with a sealing sheet containing an anti-fluorescence quenching agent. After the nuclei were stained with DAPI, blue fluorescence was displayed, and the apoptotic cells were stained with the TUNEL reaction mixture to show green fluorescence.

### Statistical analysis

All data were analyzed using GraphPad Prism 6.0 software. The results are expressed as the mean ± SD. The differences between each group of values and its control group were evaluated by a two-sided Student’s *t*-test. The differences in three or four groups were analyzed by ANOVA followed by Tukey’s post hoc test. *P* < 0.05 was considered statistically significant. *P*-value representations are indicated as follows: *P < 0.05.

## Supplementary information

Supplementary figure legends

Supplementary figure 1

Supplementary figure 2

Supplementary figure 3

Supplementary figure 4
