# Supplementary material for: BMP4 overexpression induces the upregulation of APP/Tau and memory deficits in Alzheimer’s disease
Source: Cell Death Discov. 2021 Mar 15;7:51. doi: 10.1038/s41420-021-00435-x (PMC7961014; doi:10.1038/s41420-021-00435-x)
Supplement: Supplementary file 1 — Supplementary figure legends [file 41420_2021_435_MOESM1_ESM.docx]

**Supplementary figure legends**

**Supplementary figure 1:**

**A.** RT-PCR analysis was used to measure NSE-BMP4 transgene expression in adult transgenic forebrain.

**B.** The immunofluorescence assay was utilized to detect that BMP4 overexpression affected hippocampal neurogenesis.

**Supplementary figure 2:**

**A.** Left panel: Western blotting analysis was performed to measure the expression of APP and PSEN-1 in N2A cells transfected with BMP4 overexpression plasmid. Right panel: Quantitative results are illustrated for left panel. *P<0.05 vs pcDNA3.1.

**B.** Left panel: Western blotting analysis was performed to measure the expression of T-TAU, P-Thr181 TAU and P-Thr231 TAU in N2A cells transfected with BMP4 overexpression plasmid. Right panel: Quantitative results are illustrated for left panel. *P<0.05 vs pcDNA3.1.

**Supplementary figure 3:**

**A.** Left panel: Western blotting analysis was performed to measure the expression of APP and PSEN-1 proteins in N2A cells transfected with BMP4 siRNAs. Right panel: Quantitative results are illustrated for left panel. *P<0.05 vs NC. NC: nonspecific control.

**B.** Left panel: Western blotting analysis was performed to measure the expression of T-TAU, P-Thr181 TAU and P-Thr231 TAU in N2A cells transfected with BMP4 siRNAs. Right panel: Quantitative results are illustrated for left panel. *P<0.05 vs NC.

**Supplementary figure 4:**

A**.** Left panel: Western blotting analysis was performed to measure the expression of BAX and Bcl-2 in N2A cells transfected with BMP4 overexpression plasmid. Right panel: Quantitative results are illustrated for left panel. *P<0.05 vs pcDNA3.1.

**B.** Left panel: Western blotting analysis was performed to measure the expression of BAX and Bcl-2 in N2A cells transfected with BMP4 siRNAs. Right panel: Quantitative results are illustrated for left panel. *P<0.05 vs NC.
