# Supplementary figures and images for: BMP4 overexpression induces the upregulation of APP/Tau and memory deficits in Alzheimer’s disease
Source: Cell Death Discov. 2021 Mar 15;7:51. doi: 10.1038/s41420-021-00435-x (PMC7961014; doi:10.1038/s41420-021-00435-x)

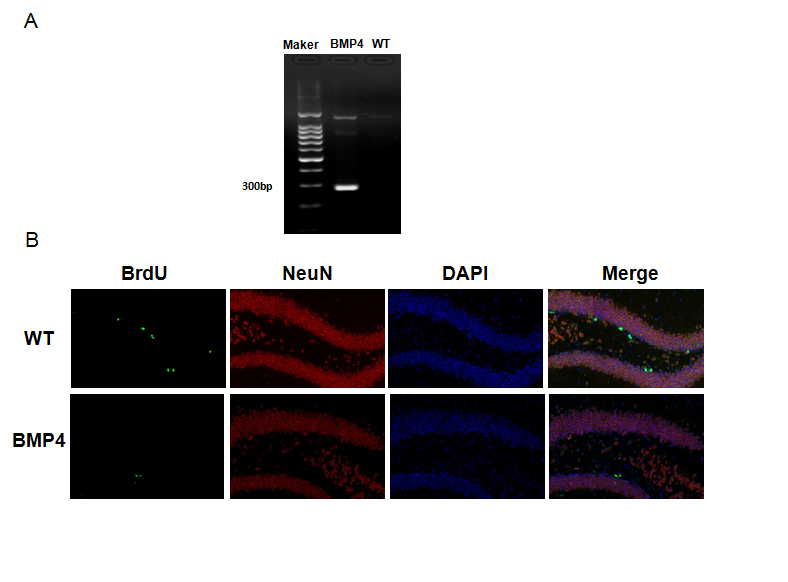

Supplement: Supplementary file 2 — Supplementary figure 1 [file 41420_2021_435_MOESM2_ESM.tif]

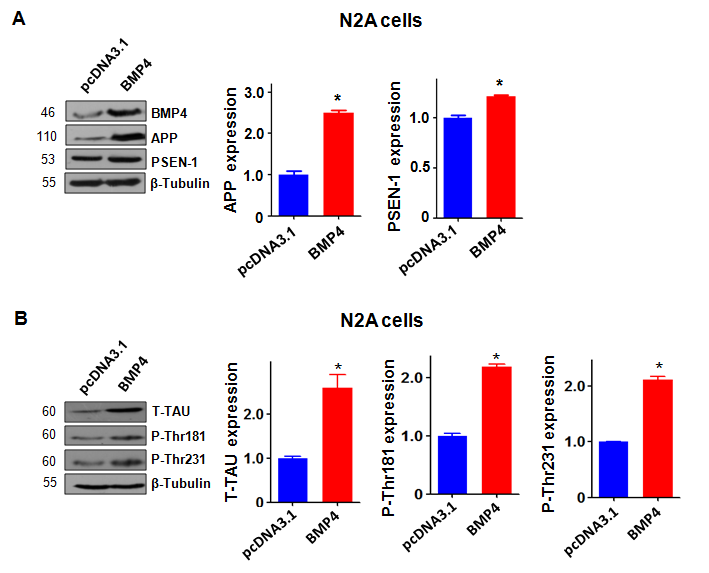

Supplement: Supplementary file 3 — Supplementary figure 2 [file 41420_2021_435_MOESM3_ESM.tif]

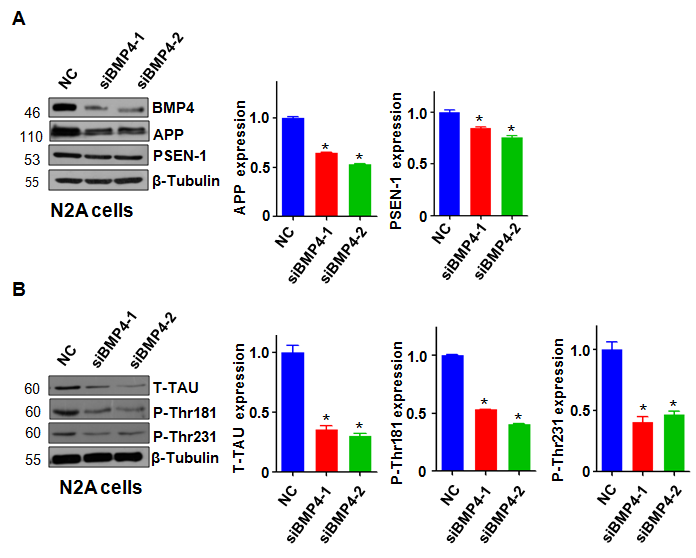

Supplement: Supplementary file 4 — Supplementary figure 3 [file 41420_2021_435_MOESM4_ESM.tif]

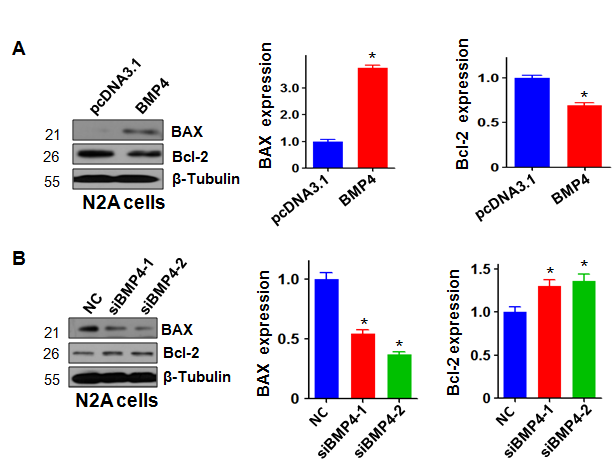

Supplement: Supplementary file 5 — Supplementary figure 4 [file 41420_2021_435_MOESM5_ESM.tif]
